# Outbreaks of clinical toxoplasmosis in humans: five decades of personal experience, perspectives and lessons learned

**DOI:** 10.1186/s13071-021-04769-4

**Published:** 2021-05-19

**Authors:** Jitender P. Dubey

**Affiliations:** United States Department of Agriculture, Agricultural Research Service, Animal Parasitic Diseases Laboratory, Beltsville Agricultural Research Center, Building 1001, Beltsville, MD 20705-2350 USA

**Keywords:** *Toxoplasma gondii*, Ocular, Outbreaks, Meat, Oocysts, Humans, Worldwide

## Abstract

**Background:**

The protozoan parasite *Toxoplasma gondii* has a worldwide distribution and a very wide host range, infecting most warm-blooded hosts. Approximately 30% of humanity is infected with *T. gondii*, but clinical toxoplasmosis is relatively infrequent. Toxoplasmosis has a wide range of clinical symptoms involving almost all organ systems. In most persons that acquire infection postnatally, symptoms (when present) are mild and mimic other diseases such as flu, Lyme disease, Q fever, hematological alterations, or mumps. It is likely that clinical disease is more common than reported. The ingestion of infected meat or food and water contaminated with oocysts are the two main modes of postnatal transmission of *Toxoplasma gondii*. The infective dose and the incubation period of *T. gondii* infection are unknown because there are no human volunteer experiments.

**Methods:**

Here, I have critically reviewed outbreaks of clinical toxoplasmosis in humans for the past 55 years, 1966–2020. Information from oocyst-acquired versus meat-acquired infections was assessed separately.

**Results:**

Most outbreaks were from Brazil. There were no apparent differences in types or severity of symptoms in meat- versus oocyst-acquired infections. Fever, cervical lymphadenopathy, myalgia, and fatigue were the most important symptoms, and these symptoms were not age-dependent. The incubation period was 7–30 days. A genetic predisposition to cause eye disease is suspected in the parasites responsible for three outbreaks (in Brazil, Canada, and India). Only a few *T. gondii* tissue cysts might suffice to cause infection, as indicated by outbreaks affecting some (but not all) individuals sharing a meal of infected meat.

**Conclusions:**

Whether the high frequency of outbreaks of toxoplasmosis in humans in Brazil is related to environmental contamination, poor hygiene, socioeconomic conditions, or to genotypes of *T. gondii* needs investigation.

**Graphic Abstract:**

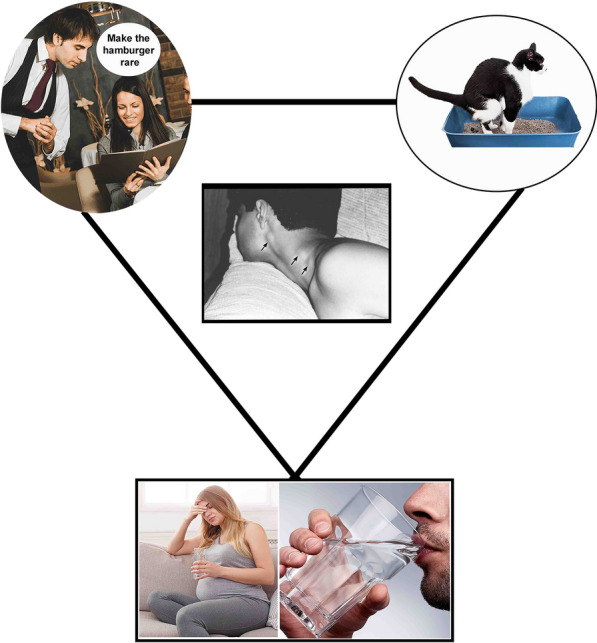

## Background

*Toxoplasma gondii* infection is widespread among humans, and its prevalence varies widely from place to place [1–5]. Approximately 30% of humanity is infected with *T. gondii*, but clinical toxoplasmosis is relatively infrequent [6, 7]. In most persons, symptoms (when present) are mild and mimic other ailments such as flu, Lyme disease, Q fever, hematological alterations, and mumps. Therefore, it is likely that in many persons these symptoms are missed, or the diagnosis is delayed [8, 9]. For example, in an outbreak of confirmed clinical toxoplasmosis in 35 persons associated with a riding stable in the USA, the diagnosis of toxoplasmosis was missed by 22 of 25 physicians [10].

The ingestion of undercooked meat containing *T. gondii* tissue cysts or ingestion of water and food contaminated with oocysts excreted by cats are the two major sources of infection. It is not known whether the severity of clinical toxoplasmosis in humans is related to the ingestion of infected meat or food contaminated with oocysts. To my knowledge, there are no volunteer experiments documenting the incubation period or infective dose by natural routes in humans. There is ample documentation of clinical toxoplasmosis in laboratory-acquired *T. gondii* infections, including attempts at suicide, but these are because of accidental inoculation by tachyzoites [11]. Based on experiments in laboratory animals, oocyst-induced infections cause more severe disease than the ingestion of tissue cysts or bradyzoites [2].

Data collected from outbreaks of clinical toxoplasmosis in humans, where many persons become ill at the same time, can provide useful information concerning the infectious stage (oocyst versus tissue cysts), incubation period, and clinical spectrum.

Until 1970, when the transmission via oocysts was recognized, ingestion of infected meat was the only mode of postnatal transmission of *T. gondii* known at that time. For the past five decades, many outbreaks of clinical toxoplasmosis in humans have been reported [12–14]. A recent report searched worldwide outbreaks of human toxoplasmosis reports from 1960 to early 1980 [14]. A geographic distribution of outbreaks was provided. Of 34 outbreaks, meat was implicated in 16, oocysts in 15, and consumption of contaminated vegetables in two instances [14].

I have been involved as an investigator or with personal knowledge of many of these outbreaks. Here, I summarize some of these outbreaks and indicate that the severity of toxoplasmosis and symptoms are probably not related to the stage ingested (oocyst versus tissue cysts), and discuss the epidemiology of these outbreaks and lessons I learned. In several instances, there were follow-up investigations after the initial description of the outbreak; these are summarized here to appreciate the clinical, social, and economic impact of the outbreaks.

## Oocyst-associated outbreaks

There were many outbreaks of toxoplasmosis associated with ingestion of food or water contaminated with oocysts. Not all of them provided data on the full clinical spectrum. Nine outbreaks with the relative frequency of various manifestations are summarized in Table 1. Some of the adult patients were ill enough to be admitted to the hospital. Several aspects of these episodes are epidemiologically and biologically interesting and therefore recalled here.

**Table 1:** Frequency of symptoms in outbreaks of toxoplasmosis in humans associated with ingestion of food or water contaminated with oocysts

Location	São Paulo, Brazil	Georgia, USA	Panama	Paraná, Brazil	São, Paulo, Brazil	Goiás, Brazil	Paraná, Brazil	Paraná, Brazil	French Guiana
References	[15]	[10, 16–18]	[19]	[20–22]	[23]	[24]	[25]	[26]	[27]
Year of outbreak	1966	1977	1979	2001	2009	2015	2015	2016	2017
No. of patients	99	35^a^	31^b^	155^c^	11^d^	14	54^e^	20^f^	20^ g^
Incubation period (days)		4–21	5–16	NR	NR	NR	NR	NR	NR
Symptoms									
Fever	79.7	94.2	90.3	82.5	81.8	85.7	51.8	NR	75.0
Lymphadenopathy	61.6	88.5	77.4	75.4	63.6	42.9	40.7	80.0	30.0
Headache	32.3	88.5	77.4	87.0	63.6	64.3	31.5	85.0	30.0
Myalgia	10.1	62.8	67.7	80.0	54.5	50	NR	75.0	5.0
Stiff neck	NR	57.1	54.8	NR	NR	NR	NR	NR	NR
Anorexia	NR	57.1	NR	69.0	54.5	71.4	NR	NR	NR
Sore throat	17.1	45.7	NR	NR	27.2	NR	NR	NR	NR
Arthralgia	4.0	25.7	29.0	61.2	36.3	57.1	NR	NR	NR
Rash	NR	22.8	0	7.0	NR	14.3	NR	NR	5.0
Confusion	3.0	20.0	NR	NR	NR	NR	NR	NR	NR
Earache	NR	17.1	NR	NR	NR	NR	NR	NR	NR
Nausea/vomiting	NR	17.1	36.6	38.7	27.2	50	NR	100	NR
Eye pain	NR	NR	25.8	NR	NR	NR	7.4	NR	NR
Blurred vision	NR	NR	NR	NR	NR	NR	22.2	NR	NR
Ophthalmic	NR	0	NR	NR	18.1	57.2	NR	15.0	5.0
Abdominal pain	NR	11.4	54.8	NR	9.0	42.9	NR	NR	NR
Asthenia	52.5	NR	NR	NR	81.8	78.6	50.0	45.0	NR
Night sweats	NR	NR	NR	53.5	81.8	42.9	NR	NR	NR
No. of abortions/congenital infection	NR	1	NR	6	NR	1	NR	5	1

NR = Not reported by the patient or not recorded

^a^Thirty-five of 37 persons were sick; they were young adults (mean age 23.8 years), 32 male, five female (three pregnant, one with congenitally infected fetus). Additional symptoms were chest pain in three, hepatitis in four, rhinitis in five of 35 patients. One person developed ocular lesions 4 years later (see text)

^b^Thirty five of 98 soldiers from one battalion were ill; three were sick enough to be hospitalized and 18 took medical leave of absence from work. Clinical toxoplasmosis was confirmed in 31. In addition to the symptoms listed above, two had hepatomegaly, one had splenomegaly, and one had chills

^c^Additionally, 128 persons had malaise. Ophthalmic examinations were conducted as a follow-up (see text). Ten women seroconverted during pregnancy; one of these had a spontaneous abortion. Six babies were born with congenital toxoplasmosis; one died at 9 months of age despite anti-*T. gondii* therapy (see text)

^d^Dysuria in two, diarrhea in two, irritability in one of 11 patients

^e^The subjects were 16 to 64 years old and the outbreak lasted 4 months. Ingestion of salad contaminated with oocysts was linked epidemiologically, but the direct cause could not be established

^f^Six (30%) persons had cough, and 15 (45%) had apathy

^g^Twenty of 54 cases had serological evidence of acute toxoplasmosis in inhabitants of an Amerindian village. Of these 20, six were adults and 14 were children, all symptomatic. A woman diagnosed at 10 weeks of pregnancy had a spontaneous abortion at 19 weeks of gestation, despite treatment with spiramycin. *T. gondii* was isolated from fetal tissues and from the blood of a 2-year-old boy

### Atlanta, Georgia, USA

In October 1977, 35 of 37 patrons (young adults) of a stable in the outskirts of Atlanta, Georgia, USA had clinical toxoplasmosis (Table 1). The stable had an outdoor and indoor riding arena. The floor of the arena was covered with fine silt and often bulldozed. Most affected persons (24 of 37) used the riding stable daily. This outbreak would have gone unreported had it not been for the keen interest of one patient who had connections with the Centers for Disease Control and Prevention (CDC) Atlanta staff (personal knowledge). Twenty-five patients independently saw their physicians; only three correctly diagnosed the symptoms as toxoplasmosis. Eventually, the CDC staff conducted an extensive case–control study. One remarkable finding was the extent of the clinical spectrum of symptoms and signs documented for the first time for toxoplasmosis in many persons (Table 1). Three of the five women were pregnant, and one had an induced abortion because she was in her first trimester; viable *T. gondii* was isolated from her fetus [16, 17].

All 37 patients were offered free eye examination and were advised to consult their doctor in the future for any ophthalmic problems. At the time of the outbreak, none of the 37 patients examined by ophthalmologists had ocular lesions. Four years later, ocular lesions were detected in one of 28 subjects [18]. The patient with eye disease was a 10-year-old girl at the time of the outbreak. She had a high antibody titer (1:4096) in both IgG and IgM immunofluorescent antibody tests. Her IgM antibody titer became negative in multiple tests, but IgG was persistent, supporting evidence for acutely acquired infection.

An epidemiological investigation suggested that patrons acquired *T. gondii* from oocysts perhaps aerosolized during the riding activity, although attempts to isolate oocysts from 29 samples of soil, sand, and sawdust from different parts of the stable were unsuccessful [17]. Attempts were made to isolate *T. gondii* from animals trapped in and around the stable. Viable *T. gondii* was isolated from tissues of two of four kittens, three of three adult cats, and four of four mice trapped in the stable in November 1977 (sample 1) but not from 12 mice, three rats, and four cotton rats trapped in the stable in December 1977 (sample 2). Bulldozing of the arena between the collection of samples 1 and 2 might have contributed to differences in results obtained with these two samples.

An unusual aspect of the outbreak was a very high rate (95%) of clinical toxoplasmosis in exposed persons. It was also the first documented outbreak linked to inhalation or ingestion of oocysts. With respect to the pathogenicity of the *T. gondii* isolates, an isolate from one of the cats and the isolate from the human fetus were highly virulent for outbred Swiss Webster mice; tenfold dilutions of *T. gondii*, estimated to contain one tachyzoite, one bradyzoite, and one oocyst, were lethal to mice. In other words, all infected mice died, irrespective of dose [17]. These results indicated that asymptomatic animals could harbor mouse-virulent *T. gondii*. At that time, there were no genetic markers available, and those isolates were not cryopreserved. Results also indicated that timing of sampling is important in an epidemiological investigation, because different results were obtained with samples 1 and 2.

*Lesson learned*: Lack of awareness hindered correct diagnosis of clinical toxoplasmosis by physicians because symptoms are nonspecific.

### Panamanian outbreak

The Panamanian outbreak occurred in 1979 in 600 soldiers of the United States Army that were on a 3-week training course in the Panama Canal area [19]. Thirty-five of 98 soldiers in one battalion became ill, and symptoms, involving many organ systems, were confirmed by diagnosis in 31 and are listed in Table 1. They ate canned food but drank water obtained locally from a small stream consisting of a series of semi-stagnant pools. The outbreak was linked to drinking water from this source although the water had been treated with iodine tablets. Although the paper did not specify the contents of these tablets, iodine tablets are commercialized for emergency purification of water from an unknown source; these are considered effective against bacteria and *Giardia lamblia*.

*Lesson learned*: Ponds in jungles can be contaminated with feces of infected wild felids. Treating water with iodine or chlorine will not kill *T. gondii* oocysts.

### Canadian outbreak

An outbreak of toxoplasmosis in humans in a western Canadian province, Greater Victoria, British Columbia was epidemiologically linked to oocyst contamination of a municipal water supply [28–30]. Between 2894 and 7718 persons were considered to have acquired *T. gondii* infection. The outbreak was suspected because many serologically positive women were identified in a short time through a screening program for pregnant women. Among these 100 cases of serological evidence of recently acquired infection, 37 were women identified through a routine perinatal screening program. The remaining 63 were identified because they sought medical attention. How many of these 100 persons had clinical symptoms of toxoplasmosis is not very clear; therefore these data are not in Table 1. Fifty-one were reported to have lymphadenopathy but it is not clear whether this figure is out of 63 symptomatic patients or includes all 100 persons. The most striking clinical feature of this outbreak was the occurrence of ocular toxoplasmosis in 20 of the 100 patients. All had unusually high levels of IgG, IgM, and IgE antibodies, which had not previously been described in groups of patients with acute toxoplasmosis retinitis. Eight patients had generalized toxoplasmosis with symptoms of night sweats, fever, chills, and headaches There were 12 additional congenitally infected children born to women who had acquired *T. gondii* during pregnancy; these were in addition to the 100 cases. Six of these had retinal lesions, three of which were bilateral. Serotyping based on peptides present in serum samples from four congenitally infected children from this outbreak had non-clonal type II *T. gondii* strains [31].

Epidemiological investigation indicated that the source of outbreak was the water supplied from the relatively small Humpback Reservoir that received water from two watersheds and another reservoir; these were open, with access to cats. Heavy rainfall probably contributed to the runoff of water into the Humpback Reservoir. Water from this reservoir was not filtered but was chlorinated. Three months after the outbreak, attempts to find oocysts in water from this reservoir were unsuccessful [30]. Although oocysts were not identified in the municipal reservoir, runoff from soil contaminated with feces of infected domestic cats or cougars was considered the likely source. Four of seven domestic cats (*Felis catus*) trapped from water reservoir sites were seropositive and most likely already excreted oocysts. Twelve cougars (*Felis concolor*) from Vancouver Island were euthanized and tested for *T. gondii* infection; 11 of 12 were seropositive, and 12.5 million viable *T. gondii* oocysts were isolated from the rectal feces taken from a single cougar. *Toxoplasma gondii* oocysts were also found in fecal scats believed to be from the same cougar, whose rectal contents had oocysts [32–34]. The *T. gondii* strain from the cougar has been fully sequenced and belongs to ToxoDB genotype #66, haplogroup 11 [35].

*Lessons learned*: Some strains of *T. gondii* may cause more eye disease than others. The environment is heavily contaminated with oocysts, and the control of domestic and wild populations of cats is not occurring. Proper sedimentation and filtration of municipal water can remove most *T. gondii* oocysts. The Canadian outbreak occurred because of a breakdown in sedimentation procedures. Chlorination will not kill *T. gondii* oocysts.

### Indian outbreak

A most unusual outbreak of ocular toxoplasmosis occurred in residents of the South Indian city of Coimbatore, state of Tamil Nadu [36, 37]. The patients were 12–73 years old and were examined at the Aravind Eye Hospital Postgraduate Institute of Ophthalmology. The outbreak peaked in 2004; very few cases of *T. gondii* retinitis were seen in this eye clinic in years before and after this period. A total of 248 patients had ocular disease. Evidence for the acquired toxoplasmosis was as follows: (i) most patients had high IgM and IgG antibodies, (ii) the retinal lesions were recently acquired and old scars were absent, and (iii) lesions were mostly unilateral. Most patients were residents of Coimbatore and its suburbs with minimal immigration from other regions of India. Water was considered the source of infection because of the demographics; the short period of the outbreak and patients were mostly unrelated to each other thus discounting sharing common meat meals. The city was supplied by water from the Siruvani Reservoir and the catchment area is likely to be exposed to felids. A heavy rainfall period preceding the outbreak might have contributed to the outbreak.

*Lessons learned*: Some strains of *T. gondii* may be predisposed to causing ocular disease.

### Turkish outbreak

This outbreak of acute toxoplasmosis occurred in school children in Turkey [38]. In 2002, a boarding school in Izmir saw 171 (9.5%) of 1797 students, aged 14–18-years old, develop mild flu-like illness. All students were examined physically, including ophthalmic testing. The symptoms were typical of acquired toxoplasmosis (cervical lymphadenopathy, fever, myalgia, headache, and dizziness). None of the students had ocular lesions. Antibodies to *T. gondii* were found in all 171 students by means of several serological techniques; all were positive for IgM antibodies, and 40 of 43 randomly selected students also had low-avidity *T. gondii* antibodies; IgM and low-avidity antibodies are indicative of recent infection. Epidemiological investigation revealed no common source. Near the dining hall, there was a sheltering place for large numbers of stray cats. However, the school authorities removed the cats before they could be tested for *T. gondii*.

*Lessons learned*: This is the largest outbreak in school children.

## Brazil, hotspot for outbreaks

### São José dos Campos, São Paulo

This outbreak is included here for historical reasons. It was one of the first outbreaks recognized before the discovery of the oocyst in 1970 [15]. The outbreak occurred in students at the university in São José dos Campos in 1966 and lasted 2 months (March–May). Ninety-nine of 500 students at the campus felt sick and were serologically tested; all of them were seropositive, most of them with high antibody titers by the dye test and indirect fluorescent antibody tests [15]. Ninety-seven of 99 had one of the symptoms listed in Table 1; most had fever, lymphadenopathy, asthenia, and headache. Although the source of the outbreak was unknown at that time, retrospectively it is most likely because of the ingestion of food/water contaminated with oocysts.

*Lessons learned*: Flu-like symptoms of toxoplasmosis can go unnoticed unless a very large number of cases are affected.

### Santa Isabel do Ivaí, Paraná

The municipality of Santa Isabel do Ivaí, Paraná has a human population of less than 10,000. A large outbreak of toxoplasmosis was reported from this town; this outbreak has been the source of great social, economic, medical concern and debate [20–22]. In November 2001, a local physician requested serological tests to diagnose dengue, mononucleosis, cytomegalovirus infection, hepatitis, and toxoplasmosis, and two persons were found seropositive for *T. gondii* IgM and IgG antibodies. By the end of 2001, 294 similar cases had been recognized, and the outbreak peaked between November 2001 and January 2002; 155 persons had symptoms of acute toxoplasmosis (Table 1). A total of 3868 persons were tested serologically for IgM and IgG antibodies. Out of these, the eyes of 454 IgM-positive individuals were examined by highly specialized ophthalmologists. Ocular lesions were seen in 33 of 288 IgM-positive persons; 13 of these had necrotizing retinitis and 17 had focal retinal whitening [21]. Most of the necrotizing lesions involved macula. New lesions were seen 1 year after the initial outbreak [21, 39]. Ten women seroconverted during pregnancy; one of these had a spontaneous abortion and three escaped congenital infection [22]. Six babies were born with congenital toxoplasmosis; one died at 9 months of age despite anti-*T. gondii* therapy. One woman had tetra-eye involvement; both of her eyes and both eyes of her infant were affected (Rubens Belfort, personal communication to J.P. Dubey). Five congenitally infected children were followed clinically for 13 years [22]. At the time of the outbreak, the mothers of these five children were in their third trimester. All five had ocular lesions. Four children underwent neurological testing; all four had learning disabilities [22].

The city was served by two underground water cisterns (A and B). Cistern A was epidemiologically associated with the outbreak. The source of water was a deep well. The cisterns were not completely sealed, and the water was not filtered by/with chlorination before human consumption. A cat living near the cistern A area delivered three kittens in October 2001. The kittens were living on the top of the water cistern and were probably weaned in early November 2001. One of these kittens was trapped and was seropositive for *T. gondii* antibodies; viable *T. gondii* was isolated from tissues of this cat euthanized on 24 January 2003 [40].

Viable *T. gondii* was isolated from water served by cistern A. The methods used and difficulties encountered for isolating *T. gondii* from water were unusual [20] and are described here. Two months into the outbreak, 4650 L of water samples were collected from small rooftop water reservoirs in four schools that were served by this reservoir. The water was filtered through Fluoropore membrane 0.2 μm filters (Millipore); these filters have laminated backing to withstand suction force. The filtration apparatus consisted of a Büchner funnel and a vacuum pump. Initially, 19 L of water was retained on filters, and the filters were processed further. The water was centrifuged, and the volume reduced to 60 mL. The filters were cut into 504 small pieces. One-third of these samples in water with membranes were shipped from Brazil to my laboratory in the Beltsville United States Department of Agriculture research center for bioassay. Laboratory-raised *T. gondii*-free pigs, cats, and mice were used for bioassay. The pigs and cats were 2 months old and had no antibodies to *T. gondii* in 1:10 dilution of serum assayed by the modified agglutination test (MAT). The filters and contaminated water were fed to pigs in pig feed. The pigs were housed in individual floor pens. The filters were not digested and were excreted with pig feces for several days. The pigs remained asymptomatic but seroconverted to *T. gondii*. They were euthanized on day 35 post-inoculation, and their pooled brain, heart, and leg muscle (100 g) were fed ad libitum to four cats; one cat for each pig. The cats excreted oocysts 5 days later. Oocysts were sporulated in 2% sulfuric acid and orally inoculated into Swiss Webster outbred albino mice. All mice became sick, and tachyzoites were found in their mesenteric lymph nodes on day 4. Lymph node homogenates were subinoculated into a group of clean mice to exclude *Hammondia hammondi* infection; these mice became sick and tachyzoites were found in their lungs on day 8 post-inoculation. The number of oocysts in the inoculum fed to pigs was unknown. Pigs are highly susceptible to *T. gondii* oocysts by the oral route; one oocyst is infective to pigs [41]. This investigation also illustrated that the strain of *T. gondii* that sickened mice and humans was nonpathogenic for pigs. Viable *T. gondii* also isolated from water collected from rooftop tanks of four schools served by cistern A was genetically characterized [42, 43]. Mice inoculated with the *T. gondii* outbreak strain were found to have the same serotype as in 13 of 20 symptomatic patients [43]. This was the first definitive demonstration of *T. gondii* oocysts in water. After the outbreak, the municipality built a new water cistern at a high elevation [22].

*Lessons learned*: A few infected cats can contaminate a large water supply, with serious social, economic, and medical consequences.

### Londrina, Paraná

Out of 772 employees at a research institution in Londrina, Paraná, 674 were tested for *T. gondii* antibodies; 73 were considered to have serological evidence of acute toxoplasmosis [25]. Fifty-four patients had symptoms (Table 1).

*Lesson learned*: The ingestion of salads served at a restaurant on-site was considered the probable cause.

### Montes Carlos de Goiás

Another outbreak in Montes Claros de Goiás in the state of Goiás was epidemiologically linked to consumption of fresh cheese from raw cow’s milk [24]. Symptoms noted are summarized in Table 1. The farm had very unsanitary conditions, and the operation was eventually suspended by state authorities.

*Lesson learned*: Contamination of cheese or milk with water contaminated with oocysts was considered the source of the outbreak.

### Santa Maria, Rio Grande do Sul

A very large outbreak was reported in the city of Santa Maria in 2018 [44–51]. The information from this outbreak reported is piecemeal, with some information only available mainly on the internet [44–47]. In total, from 2270 suspected cases, 931 were confirmed, including 146 pregnant women, 3 fetal deaths, 10 abortions, and 29 instances of congenital toxoplasmosis. Thirty-nine of 41 neighborhoods of Santa Maria city were affected. By September 2018, of the 748 persons with a confirmed diagnosis, 86% had fever, 80% had myalgia, 76% had lymphadenopathy, 8.3% were admitted to hospitals, and 32 had ocular lesions. Viable *T. gondii* was isolated from the placental tissue of two women by bioassay in mice [49]. Clinical toxoplasmosis had been diagnosed in the third trimester of gestation and the women were treated with sulfadiazine and pyrimethamine; both delivered live children [49]. An additional five women had fetal death and three aborted. Viable *T. gondii* was isolated from the placentas of all five [50]. Additionally, *T. gondii* DNA was detected by PCR in amniotic fluid, fetal tissues, and placentas [50].

The eyes of 187 infants born to 184 mothers (three mothers had twins) who were pregnant during the outbreak were examined by ophthalmologists who were specialists in ocular toxoplasmosis [51]. The mothers had serological evidence of recently acquired *T. gondii* infection. One mother with a twin pregnancy gave birth to a dead fetus and a child with congenital toxoplasmosis at 29 weeks of gestation [51]. Of these 187 infants, 29 (15.5%) had congenital toxoplasmosis and 19 (10.2%) had ocular lesions. The ocular lesions of 19 infants (38 eyes) included retinochoroiditis in 29 eyes, optic nerve abnormalities in 5 eyes, microphthalmia in 1 eye and cataract in 2 eyes. Bilateral ocular lesions were found in 10 of 19 (52.6%) infants, supporting evidence for congenital infection. Thirteen (7.2%) of 1811 infants tested had cerebral calcification. Unlike in Europe, maternal infection during the third trimester was associated with a higher rate of congenital toxoplasmosis independent of maternal treatment [51].

*Lessons learned*: This is the largest oocyst-associated outbreak. Mothers who acquired *T. gondii* infection during the third trimester had fetuses with severe toxoplasmosis.

### São Luís, Maranhão

Thirty cases of acute toxoplasmosis were epidemiologically linked to consumption of faucet-mount filtered water [52]. This is a retrospective report of an outbreak that occurred in a small town, São Luís, with a population of 130 persons resident in 46 houses. Of 90 persons serologically tested, 33 had serological evidence (IgM-positive) of acute toxoplasmosis with symptoms of lymphadenopathy, fever, and headache. The outbreak lasted 4 months (May–August 2006).

*Lessons learned*: Drinking water from a well was considered the source of oocysts for the outbreak.

### Araraquara, São Paulo

In an industrial plant, 11 of 25 persons had serological evidence of acute toxoplasmosis [23]. A wide spectrum of symptoms were present in 11 patients (Table 1). Epidemiological investigation indicated that salads and green vegetables were the most likely source of the outbreak.

*Lessons learned*: Patients had a wide spectrum of symptoms.

## Meat-borne outbreaks

Information on the few meat-borne toxoplasmosis outbreaks in the USA is summarized in Table 2.

**Table 2:** Frequency of symptoms in outbreaks of meat-borne toxoplasmosis in humans in the USA

Location	New York City	Pennsylvania	New York City	South Carolina	Tennessee	Wisconsin	Illinois
References	[53]	[54]	[55]	[56]	[57]	[9]	[8]
Year of outbreak	1968	1974	1975	1979	2017	2017	2018
No. of patients	5^a^	4^b^	5	3	2^c^	9^d^	6^e^
Meat ingested	Beef	Kibbeh nayeh (Syrian dish)	Lamb	Venison	Venison	Venison	Venison
Incubation period (days)	8–13	10–30	8–13	7–10	7	7	4–14
Patients with symptoms (%)							
Symptoms							
Fever	100	100	100	100	100	100	100
Lymphadenopathy	60.0	75.0	66.6	NR	NR	33.3	NR
Headache	100	NR	66.6	100	NR	100	100
Chills	40.0	100	NR	100	NR	100	NR
Sweats	NR	NR	NR	100	NR	100	66.6
Decreased appetite	NR	NR	NR	100	100	88.8	NR
Fatigue	NR	100	80.0	100	NR	88.8	83.3
Myalgia	80.0	NR	66.6	NR	100	NR	NR
Anorexia	40.0	NR	NR	NR	NR	NR	NR
Sore throat	NR	NR	20.0	NR	NR	77.7	NR
Arthralgia	NR	NR	20.0	NR	NR	77.7	83.3
Rash	60.0	NR	20.0	33.3	NR	NR	NR
Confusion	40.0	NR	NR	NR	NR	11.1	NR
Nausea	40.0	NR	NR	NR	NR	NR	NR
Eye pain	NR	NR	NR	NR	NR	11.1	NR
Blurred vision	NR	NR	NR	NR	NR	33.3	NR
Ophthalmic	NR	NR	20.0	NR	NR	16.6	NR
Abdominal pain	20.0	NR	NR	NR	NR	NR	NR

NR = Not reported by patient or not recorded

^a^Ate undercooked hamburgers on 5 March 1968. Two patients had abnormal sensation (dysesthesia) in lower extremities; splenomegaly in three

^b^Ate Kibbeh nayeh (a beef dish) at a wedding party in a Syrian restaurant on 23 November 1974

^c^Patient and his adult grandson ate pan-seared heart of deer; both of them developed identical symptoms 7 days later

^d^Ate undercooked grilled venison kabobs at a deer retreat on 29 September 2017

^e^Canadian hunters ate venison at a retreat in Illinois, 22 November–4 December 2018

Among these outbreaks, the most definitive information is from the episode in New York City, USA, because the date meat consumed was known, the subjects were medical students who could better describe their symptoms, and the investigators were experts on toxoplasmosis [53]. Five medical students developed symptoms of acute toxoplasmosis characterized by headache, fever, lymphadenopathy, myalgia, and splenomegaly during the second week after eating rare beef hamburgers at a university snack bar [53]. These five students were unrelated and lived and ate separately, except on the evening of 5 March 1968, when they were in a hurry to attend a talk by a famous heart surgeon (Dr. Christiaan Barnard, who performed the first heart transplant), and the hamburgers were made in a hurry at the university cafeteria. Fortunately, only one of them was female, and she was not pregnant. Whether the ground beef used to make hamburgers was contaminated with pork or lamb could not be ascertained, but seems likely. The outbreak was quickly diagnosed because of the personal interest of Dr. Kean (the lead author), who was on the faculty of medicine and specialized in toxoplasmosis. In the previous 5 years, only two cases of acquired toxoplasmosis had been diagnosed in medical students at his institution [53]. Unlike many other reports, it was not a retrospective investigation.

Four other outbreaks from the USA listed in Table 2 were linked to eating infected venison. Additionally, ocular toxoplasmosis was diagnosed in five patients with flu-like symptoms in Michigan, USA; all patients recalled eating undercooked venison, but there was no link to a common meal and the patients lived separately [58]. In the two most recent episodes of acute toxoplasmosis in deer hunters related to consumption of undercooked fresh venison, diagnosis and treatment was delayed because of symptoms mimicking Lyme disease, Q fever, and hematological alterations [8.9]. In the Wisconsin outbreak, portions of frozen muscle from the hunted deer that were consumed by the attendees were tested for *T. gondii* by PCR. Viable *T. gondii*, however, was not isolated by bioassay in mice, probably because the sample had been frozen. Genetic characterization of DNA from venison by 15 microsatellite testing revealed the strain was atypical, most aligned to TOXODB genotype #5, belonging to haplogroup 12 [9]. *Toxoplasma gondii* infections are common in white-tailed deer (WTD) in the USA. In a recent nationwide survey, *T. gondii* antibodies were detected in 329 of 912 (36%) and viable *T. gondii* was isolated from 36 WTD [59]; 24 of the 36 (66.6%) isolates were haplogroup 12, commonly found in wildlife in the USA. It is worth noting that wildlife *T. gondii* strains can cause clinical toxoplasmosis in humans. Recently, seven adult (54–71 years old) patients in the USA developed ocular toxoplasmosis after eating rare deer meat or dressing hunted deer; they had experienced headaches and fever before development of ocular symptoms [60].

Data from meat-borne outbreaks from other countries are summarized in Table 3.

**Table 3:** Frequency of symptoms in outbreaks of meat-borne toxoplasmosis in humans in Australia, Brazil, France, and the United Kingdom

Location	Paraná, Brazil	Brisbane, Australia	Kyongsangbuk-do, South Korea, Korea	Rio Grande do Sul, Brazil	São Paulo, Brazil	Aveyron, France	London, United Kingdom
References	[61]	[62]	[63]	[64]	[65]	[66]	[67]
Year of outbreak	1993	1994	1995	2005	2006	2010	NR
No. of patients	17^b^	12^a^	5^e^	10^c^	6^d^	5	3
Meat ingested	Lamb	Kangaroo meat	Pork	Pork	Beef	Lamb roast	Lamb
Incubation period (days)	6–13	3–25 (average 11)	30	6–23	1–8	30	8
Patients with symptoms (%)							
Fever	94.1	50.0	NR	70.0	100	80	100
Lymphadenopathy	94.1	66.6	100	80.0	67.0	100	66.6
Headache	94.1	50.0	NR	75.0	50.0	0	100
Chills	NR	8.3	NR	NR	NR	NR	NR
Sweats	NR	33.3	NR	70.0	NR	NR	NR
Decreased appetite	NR	NR	NR	NR	NR	0	NR
Fatigue	NR	16.6	NR	NR	NR	80.0	NR
Myalgia	94.1	58.3	NR	80.0	83.0	80.0	100
Anorexia	NR	NR	NR	NR	NR	0	NR
Sore throat	NR	25.0	NR	NR	NR	NR	NR
Arthralgia	94.1	NR	NR	NR	67.0	NR	NR
Rash	11.8	8.3	NR	NR	17.0	NR	NR
Confusion	NR	NR	NR	NR	NR	0	NR
Nausea	NR	NR	NR	NR	NR	NR	NR
Eye pain	NR	NR	NR	NR	NR	0	NR
Blurred vision	NR	NR	NR	NR	NR	0	NR
Ophthalmic	5.8	8.3	NR	10.0	NR	0	NR
Abdominal pain	NR	NR	NR	NR	NR	NR	NR

NR = Not reported by patients or not recorded

0 = no symptoms

^a^Sixty persons attended a Christmas party. A variety of foods were served, but kangaroo meat was suspect because it was served rare. One woman with acute toxoplasmosis had a congenitally infected baby. Two patients had altered liver functions and atypical lymphocytosis

^b^Symptoms lasted 1–10 days. Ten (62.5%) of 16 patients had fever higher than 39.5ºC from 6–9 days. One patient developed chorioretinitis 20 days after onset of symptoms. Other findings were hepatomegaly in 35.3% and splenomegaly in 23.5%

^c^Possible ingestion of processed pork loin during a party. One patient had ocular lesions and vision loss 3 months after the first symptoms

^d^At a party, ingestion of raw ground beef was associated with *T. gondii* infection; six of 10 people who ingested beef became infected, while four who did not ingest were not infected. Hepatomegaly was diagnosed in 33.0%. A mother (30 weeks of pregnancy) delivered a dead fetus despite treatment with spiramycin, sulfadiazine, and pyrimethamine

^e^On 1 January 1995, five of eleven 20-year-old Korean soldiers feasted on raw liver and meat of a pig reared in a military compound. All suddenly developed lymphadenopathy within 1 week beginning 31 January. The diagnosis was confirmed by serological testing by multiple antibody test. The six persons without symptoms were seronegative

The first toxoplasmosis outbreak linked to eating undercooked pork occurred in South Korea [63]. In two separate incidents, eight adults developed lymphadenopathy and ocular disease after feasting on raw or undercooked pork; one of them became blind despite chemotherapy [63]. In the first episode, three adult merchants developed unilateral retinochoroiditis after eating a meal consisting of the raw spleen and liver of a wild pig on 27 September 1994. All three patients developed ocular disease at about the same time. Patient 1 became totally blind. In the second outbreak, five soldiers developed lymphadenopathy 7 days after feasting on raw liver and uncooked meat from a domestic pig at a New Year’s party on 1 January 1995, and these data are summarized in Table 2. Lymph nodes in the neck, axilla, and chest area were enlarged; no other symptoms were noted. Six other soldiers who also feasted on the pig at the same time remained seronegative and healthy. I learned of this outbreak in 1995 during my visit to the School of Medicine, Catholic University of Korea, Seoul, but the diagnosis had not been confirmed at that time. I brought the archived sera to the USA for detailed serological testing in Dr. Jack Remington’s laboratory at Stanford, California. Sera were tested for *T. gondii* antibodies by the modified agglutination test (both formalin-fixed and acetone fixed antigens), dye test, IgM and IgG ELISA, and the latex agglutination test. All 8 patients had evidence of recently acquired infection.

The outbreak in Australia needs a comment for several reasons. The outbreak occurred among 60 people who attended a party in Brisbane in November 1994 [62]. Acute toxoplasmosis was diagnosed retrospectively in at least 12 persons (Table 3). This report has been rarely cited because it was published in a communicable disease bulletin and not a regular periodical with a wider circulation [62]. It was also the first documentation of toxoplasmosis associated with eating undercooked kangaroo meat. The outbreak was recognized because of accidental diagnosis of congenital toxoplasmosis in a child in the index case. An amniocentesis was performed for possible Rhesus incompatibility (RH factor) when the mother was in the 34th week of gestation. One week later she delivered a baby because of RH factor. At 3 months of age, *T. gondii* chorioretinitis was discovered in the infant. The mother’s medical records and testing of archived sera revealed that she serologically converted during pregnancy, and she recalled eating undercooked kangaroo meat at the party when she was 28 weeks pregnant. She also recalled having myalgia and lethargy 3 weeks after the party. She was also aware of a second woman who saw a physician because she developed fever, nausea, myalgia, and arthralgia 9 days after the meal at the party. Subsequent inquiries revealed 10 additional cases of acute toxoplasmosis (Table 3). In conclusion, this outbreak was discovered because of the fetus being tested for RH factor.

## Multifactorial outbreaks in French Guiana and Suriname

Two outbreaks of toxoplasmosis occurred in French Guiana and Suriname [27, 68]. Unusual aspects of these outbreaks were as follows: (i) Both outbreaks occurred in small remote villages with a small population. (ii) Both were associated with mortality. (iii) Mouse-virulent *T. gondii* isolates were obtained from human tissues. (iv) Although a clear source of infection was not linked to the outbreak, ingestion of oocysts was the most likely cause. (v) Both outbreaks were investigated by the same research group. (vi) There was no evidence of HIV or other immunosuppressive diseases. (vii) There was no electricity or water supply. Each house had a rainwater reservoir. Hunting and fishing provided most of the meats consumed. (viii) Domestication of cats probably contributed to these outbreaks.

### Suriname

The outbreak occurred in 2004 in the village of Patam, located near the French Guiana border, and surrounded by Amazonian forest [68]. One Maroon tribe family with 34 members lived in the village. Five persons were hospitalized. Of 31 persons tested, seven had clinical toxoplasmosis; of the 24 who were asymptomatic, only three had *T. gondii* antibodies. One 56-year-old female died of acute toxoplasmosis despite treatment. Two women were pregnant. One 6-day-old child and one fetus at 22 weeks gestational age died of acute toxoplasmosis. Viable *T. gondii* strains designated GUY-2004-ABE, GUY-2004-ANG, GUY-2004-ANG1, GUY-2004-TER, and GUY-2004-TER1 were isolated from peripheral blood of four patients (the 56-year-old female, one 21-year-old mother, one 22-year-old female, and an 8-month-old male) and tissues of the dead expelled fetus [68].

*Lessons learned*: *T. gondii* can cause fatal toxoplasmosis in adult immunocompetent persons, and human virulent isolates are circulating in Amazonian jungles.

### French Guiana

This outbreak occurred in Camopi, a remote Amerindian village along the Oyapock River, French Guiana in 2017 following an episode of warm weather and heavy rains [27]. The village has 1800 persons. Initially, two adults and two of their children sought medical attention for febrile illness. Eventually, acute toxoplasmosis was diagnosed in 20 of 54 persons tested (Table 1). The 54 persons were from homes with one of the patients. Fever, lymphadenopathy, and cough were predominant symptoms (Table 1). Four patients (two adults and two of their children) were hospitalized but all recovered. A woman infected at 10 weeks of gestation was medicated with spiramycin but her fetus died in utero at 19 weeks of gestation. Viable *T. gondii* (GUY070-KEL) was isolated from fetal tissues. Viable *T. gondii* (GUY066-MON) was also isolated from a 2-year-old boy, unrelated to other patients. *Toxoplasma gondii* DNA was detected from a water sample collected from one household, a soil sample of one household, and a random sample from another site [27].

*Lesson learned*: This is the largest outbreak from French Guiana. Heavy rains and warm weather probably contributed to the outbreak.

## Overall conclusions

### Clinical spectrum

There are no apparent differences in type or severity of symptoms in meat- versus oocyst-acquired infections, although statistical analysis was not performed. Fever, cervical lymphadenopathy, myalgia, and fatigue are the most important symptoms of acquired toxoplasmosis, and symptoms are not age-dependent. Diarrhea and renal ailments were rare.

### Infective dose

More than half of the people eating a common infected meat meal may escape infection [63, 67]. For example, out of 11 soldiers who feasted on raw/undercooked meat from one pig, only five became infected, whereas the other six remained seronegative and healthy [63]. These data suggest an uneven distribution and low numbers of tissue cysts in meat, confirming observations based on experimental food animals [2]. The Paraná, Brazil [20] outbreak suggested that only a few cats are required to contaminate millions of gallons of water. Considering the dilution factor, it is likely that only a few oocysts were ingested by patients.

### Eye disease and *T. gondii* genotype

The fact that a very high number of patients in the oocyst-transmitted outbreaks in Canada [29], Brazil [21], and India [37] developed ocular lesions suggests *T. gondii* genotype tropism.
